# Pedigree genome data of an early-matured *Geng/japonica* glutinous rice mega variety Longgeng 57

**DOI:** 10.1038/s41597-024-03057-x

**Published:** 2024-02-22

**Authors:** Yuanbao Lei, Yunjiang Zhang, Linyun Xu, Wendong Ma, Ziqi Zhou, Jie Li, Pengyu Quan, Muhiuddin Faruquee, Dechen Yang, Fan Zhang, Yongli Zhou, Guangjun Quan, Xiuqin Zhao, Wensheng Wang, Bailong Liu, Zhikang Li, Jianlong Xu, Tianqing Zheng

**Affiliations:** 1 Jiamusi Rice Research Institute of Heilongjiang Academy of Agricultural Sciences, Jiamusi, 154026 China; 2 Institute of Crop Sciences/State Key Laboratory of Crop Gene Resources and Breeding/National Key Facility for Crop Gene Resources and Genetic Improvement, Chinese Academy of Agricultural Sciences, Beijing, 100081 China; 3 Rice Research Institute, Guangxi Academy of Agricultural Sciences, Nanning, 530007 China; 4 Heilongjiang Lianjiangkou Seed Co., Ltd, Jiamusi, 154024 China; 5 International Rice Research Institute, Bangladesh Office, Dhaka, 1213 Bangladesh; 6 National Nanfan Research Institute, Chinese Academy of Agricultural Sciences, Sanya, 572024 China; 7 Hainan Yazhou Bay Seed Lab, Sanya, 572024 China

**Keywords:** Plant breeding, Agricultural genetics

## Abstract

By using PacBio HiFi technology, we produced over 700 Gb of long-read sequencing (LRS) raw data; and by using Illumina paired-end whole-genome shotgun (WGS) sequencing technology, we generated more than 70 Gb of short-read sequencing (SRS) data. With LRS data, we assembled one genome and then generate a set of annotation data for an early-matured *Geng/japonica* glutinous rice mega variety genome, Longgeng 57 (LG57), which carries multiple elite traits including good grain quality and wide adaptability. Together with the SRS data from three parents of LG57, pedigree genome variations were called for three representative types of genes. These data sets can be used for deep variation mining, aid in the discovery of new insights into genome structure, function, and evolution, and help to provide essential support to biological research in general.

## Background & Summary

In recent years, the planting area for rice (*Oryza sativa* L.) in Heilongjiang (HLJ) province of China has increased to around 4 million ha^1^. For this global largest planting region for early *Geng*/*japonica* rice, which is about 2.6 times larger than the rice planting area of Japan^2^, determining how to transfer its advantages in agriculture to other branches of the economy remains a significant challenge for agriculture researchers.

Early-matured *Geng*/*japonica* varieties provide the base for food security^3^, and supply critical agro-industrial materials, especially glutinous varieties. Glutinous rice, also called sticky rice, is becoming increasingly popular because of growing public awareness of health issues^4^. Glutinous rice has health benefits in managing diabetes, inhibiting chronic diseases, enhancing digestion, and reducing inflammation^5^. In addition to being an elite cooking material for a low gluten diet and ‘good food’^6^, glutinous rice also provides raw materials for environment-friendly industry^7–9^. Longgeng 57 (LG57), a glutinous early variety, has favorable quality and stable-yield behavior in the early *Geng*/*japonica* planting region; therefore, it is now planted over more than 120,000 ha per year on average.

Grain quality traits of rice are largely controlled by major genes, such as *Waxy* for the amylose content and *OsNramp5* for the mineral nutritional quality^10–12^. Thus, further improvement of grain quality of glutinous rice, e.g., LG57, also requires more genome information.

Currently, joint analysis has become a trend in biotechnology-based rice breeding in HLJ. For example, the Rice Molecular Breeding (RMB) laboratory from the Institute of Crop Science (ICS), Chinese Academy of Agricultural Sciences (CAAS), has set up a genome-based breeding scheme with the aid of both core germplasms of 3K-RG^13^, and the Rice Functional Genomics Breeding (RFGB) information platform^14^. It also widely cooperates with local research institutes from HLJ, including Jiamusi Rice Research Institute (JMS-RRI) and Suihua RRI (SH-RRI)^3^. Herein, we present a dataset from a collaboration between the RMB laboratory and JMS-RRI for early-matured *Geng/japonica* including LG57. Information based on this dataset for certain target genes, such as *Waxy* and *OsNramp5*, were also included as examples for data validation. This dataset comprises more than 770 Gb of pedigree genome data that will be useful for researches in general.

## Methods

### Plant material and library construction

**Fig. 1:**
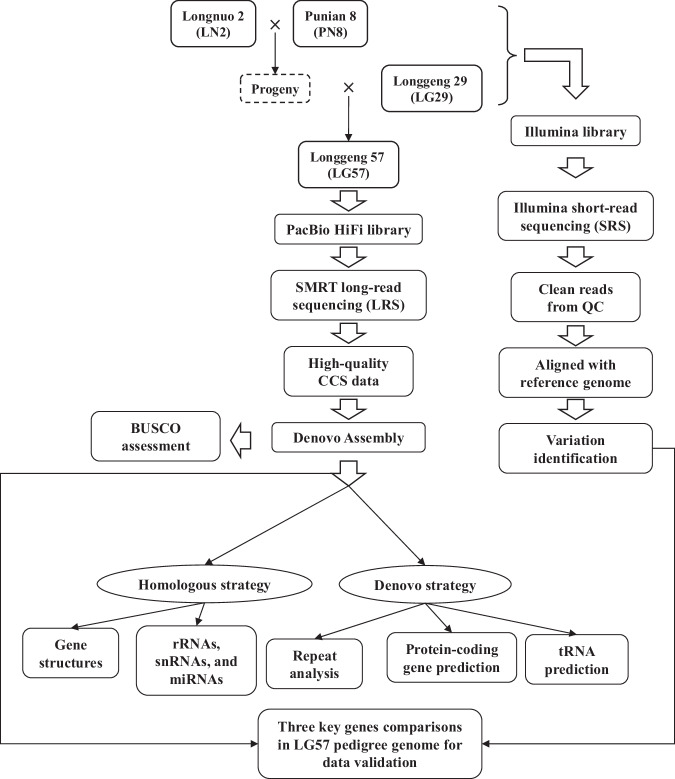
Outlines of the workflow used to generate and analyze the pedigree genome data for Longgeng 57 (LG57).

The early-matured *Geng*/*japonica* variety Longgeng57 (LG57) was developed by our own and licensed to be released in 2017 and is now one mega variety with multiple elite traits and widely planted (more than 120,000 hectare per year) in Heilongjiang province in Northeast of China. High-molecular-weight genomic DNA was extracted from 10-day-old leaves of LG57 pedigree members (multiple seeds) with modified CTAB method followed by 0.5x bead purification for twice. The DNA sample through the qualification processes by both 0.75% agarose gel assay and Nanodrop was quantified with Qubit. Then the sample of LG57 met the standard was submitted to the constructions of PacBio HiFi library for long-read sequencing (LRS). Samples of three parents (Longnuo 2 (LN2), Punian 8 (PN8), and Longgeng 29 (LG29)) were submitted to construct Illumina libraries short-read sequencing (SRS) (Fig. 1).

Genomic data were generated for all pedigree members, as listed in Table 1. Among them, PacBio (Menlo Park, CA, USA) protocols were adopted for long-read sequencing of LG57 and Illumina (San Diego, CA, USA) protocols were used for short-read sequencing. The details are as follows.

**Table 1:** Genomic data generated for pedigree of Longgeng 57.

Name	code	Tiller number	Panicle size	Genotyping method	Format	Size(Gb)
Longgeng 57	LG57	More	Smaller	PacBio	bam + fa	700 + 0.4
Longnuo 2	LN2	More	Smaller	Illumina sequencing	fq.gz	23.1
Punian 8	PN8	Fewer	Larger	Illumina sequencing	fq.gz	27.0
Longgeng 29	LG29	Fewer	Larger	Illumina sequencing	fq.gz	23.3

#### DNA sample testing

DNA extraction from samples was carried out using a routine method that met the quality standard required for sequencing according to a previous study^3^. Sample purity and quantity were detected using a Nano Photometer® (IMPLEN, Westlake Village, CA, USA) and a Qubit® 3.0 Fluorometer (Life Technologies, Carlsbad, CA, USA), respectively, in combination with Agarose electrophoresis (concentration 1%, voltage 120 V for 45 min).

#### Library construction and Inventory inspection

Covaris® g-TUBE^15^ was used to break the genomic DNA into suitable large pieces. Magnetic beads were then used for enrichment and purification. SageELF (Sage, Newcastle upon Tyne, UK) was adopted to screen and purify the DNA fragments. An Annoroad® Universal DNA Library Prep Kit V2.0 (Annoroad Gene Technology, Beijing, China) was used for sample preparation, including end repair and ligation addition.

To ensure the quality of the library, a three-step quality check procedure was adopted as follows. After the library was constructed, the Qubit 3.0 was used for preliminary quantification. Then, the library was diluted to 1 ng/μL and the insert size was checked using an Agilent 2100 instrument (Agilent, Santa Clara, CA, USA). The effective concentration of the library was accurately quantified using quantitative real-time reverse transcription PCR (qRT-PCR) in a Bio-Rad CFX96 PCR instrument with a Bio-Rad IQ SYBR GRN Kit (both Bio-Rad, Hercules, CA, USA).

#### Sequencing

The single-molecule real-time (SMRT) method was adopted for the long-read sequencing (LRS) according to standard method (PacBio). Short-read sequencing (SRS) was carried out on the NovaSeq 6000 S4 platform (Illumina) to obtain a 250 bp double-ended sequencing reads.

##### Genome assembly, validation and annotation

For the LRS data obtained by HiFi library sequencing, the raw data (subreads) from the PacBio sequencing was filtered by using SMRT link v9.0.0.92188 (https://www.pacb.com/support/software-downloads/) with default parameters to obtain high-quality circular consensus sequences (CCS) data. For the assembly, hifiasm^16^ with default parameters were employed based on the CCS data. Merqury^17^ was adopted for the quality check of LG57 assembly. Also, BUSCO (Benchmarking Universal Single-Copy Orthologs)^18^ was used for genome assembly quality assessment. BUSCO analysis with default parameters was carried out using a single-copy gene set of several large evolutionary branches based on the OrthoDB (http://cegg.unige.ch/orthodb). The gene set was compared with the assembled genome using embryophyta_odb10, and the accuracy and completeness were assessed based on the proportions and completeness of the alignment.

Based on the LG57 assembly, two strategies were adopted for genome annotation. The first was a homologous strategy. RepeatMasker with default parameters^19^ based on RepBase^20^ was used to annotate repeats. For gene structures, BLAST^21^ with evalue = 1e-5 and GeMoMa^22^ with default parameters were used. Prediction of rRNAs, snRNAs, and miRNAs was carried out by aligning the assembly with known non-coding RNA libraries, e.g., Rfam^23^.The second was a *de novo* strategy. For repeat analysis, RepeatModeler (https://www.repeatmasker.org/RepeatModeler/) with -engine ncbi was adopted. For protein-coding gene prediction, Augustus^24^ with–genemodel = partial, SNAP(https://github.com/KorfLab/SNAP), and GeneMark^25^ with default parameters were adopted. Based on the above predictions, EVidence Modeler (EVM)^26^ with default parameters was used to integrate the gene sets predicted by various strategies into a non-redundant gene set. The resulting predictive gene set was compared with various functional databases using UniProt^27^, NCBI (https://www.ncbi.nlm.nih.gov/nucleotide/), PFAM^28^, eggNOG^29^, GO (gene ontology)^30^, and KEGG (Kyoto Encyclopedia of Genes and Genomes)^31^. For tRNA sequence prediction, we used tRNAscan-SE^32^ with parameters of -X 20 and –z 8.

The SRS data were aligned to the reference genome and variations were called using a pipeline comprising BWA^33^, SAMtools^34^, and GATK^35^ with default parameters, with Nipponbare IRGSP 1.0^36^ as the reference genome.

## Data Records

The assembly of LG57 is accessible at NCBI through GenBank^37^ or the following accession ID of JAXQPT000000000^37^. Additionally, the raw read data for LG57 in the bam format are also available with accession number of SRR25376496^38^. Other sequencing pedigree genomic data for parents of LG57, including PN8 (SRR24688636)^39^, LN2 (SRR24688637)^40^, and LG29 (SRR24688635)^41^. Annotation data for LG57 are accessible through figshare^42^. All above data except for the bam files are also accessible in RFGB website (https://rfgb.rmbreeding.cn/download/publicDataDownload/download?dataset=3).

## Technical Validation

**Fig. 2:**
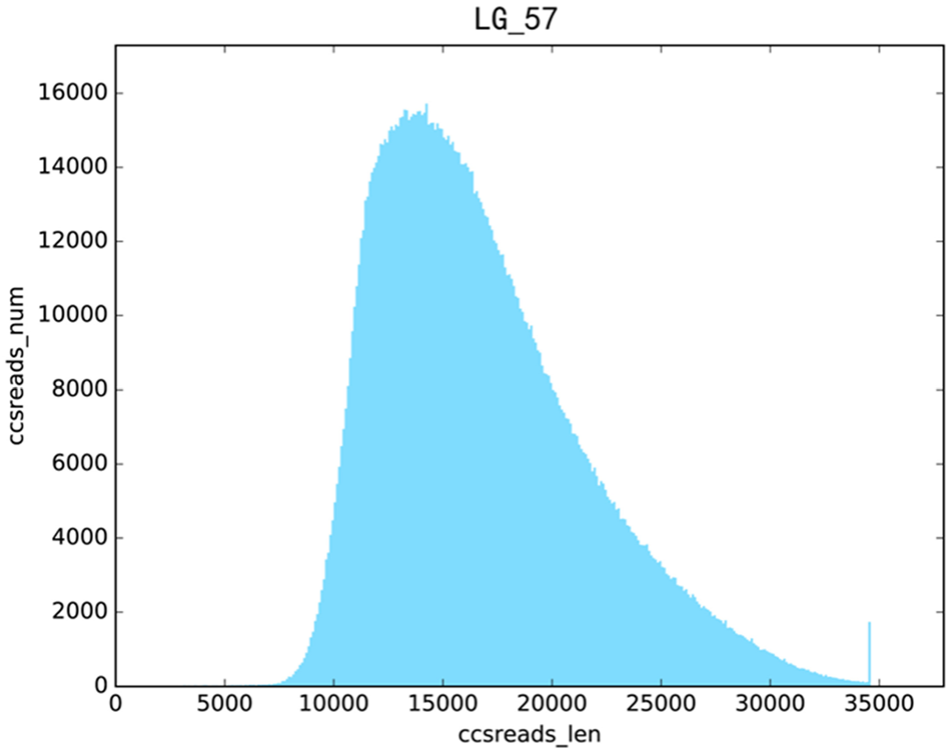
Distribution of lengths of circular consensus sequences (CCS) reads for Longgeng 57 (LG57).

A total 1,671,418 of reads were obtained. The averaged read-length is 16,831.42 bp and N50 value is more than 17 Kb. The distribution of these reads was shown in Fig. 2. A rough assembly for LG57 was carried out. A quality checking for the assembly of Longgeng 57 was also carried out by using Merqury and BUSCO. Based on the output of Merqury, the completeness of assembly was 99.5% and the QV was 62.0 (Table 2). As shown in Table 3, N50 of contig has arrived at more than 27 Mb, which is over 10 times of our previous work with SJ18^3^. As shown in Table 4, a total of 1614 groups were searched by BUSCO, the complete groups accounted for about 98.8%. Functional genes predicted in LG57 comparing with those from databases were shown in Table 5. Identified by RepeatMasker, the total length of the repeat sequences is approximately 170MB, accounting for 43.13% of the whole LG57 genome (Table 6). Prediction results of different types of non-coding RNA including miRNA, tRNA, rRNA, and snRNA were listed in Table 7. These RNAs together accounting for 81.3% of the LG57 genome. We also compared the parameters of LG57 to the other assemblies. Averaged gene length of LG57 is longer than those of the others (Table 8).

**Table 2:** Assembly quality assessment by Merqury for Longgeng 57.

Parameters	LG57
Completeness (%)	99.542
QV	62.0241
Error rate	6.27E-07

**Table 3:** Comparison of Longgeng 57 dataset with representative assemblies including mega varieties (MV) or standard references (SR).

	LG57	SJ18	MH63	Nipponbare	R498	9311	IR64
Variety types	MV (Early-matured *Geng*/*japonica*, glutinous)	MV (Early-matured *Geng*/*japonica*, aroma)	MV & SR (Three-line *Xian*/*indica* hybrid restorer)	SR (Medium-matured *Geng*/*japonica*, aroma)	SR (Three-line *Xian*/*indica* hybrid restorer)	MV & SR (Two-line *Xian*/*indica* hybrid restorer)	Mega (*Xian*/*indica*)
Total nucleotides (Mb)	392.3	418.9	359.9–395.8	373.2	390.3–423.2	426.3	367.1
N50 contig length (bp)	27,391,608	2,467,626	3,097,358 – gap free	7,711,345 – gap free	1,185,206	23,200	27,827,038
Total gene numbers	39,920	38,456	39,406	39,045	38,714	39,285	41,458

**Table 4:** Assembly quality for Longgeng 57 presented by BUSCO.

BUSCO groups searched	Number	Percentage (%)
Complete (C)	1594	98.8
Complete and single-copy (S)	1560	96.7
Complete and duplicated (D)	34	2.1
Fragmented (F)	13	0.8
Missing (M)	7	0.4
Total	1614	100

**Table 5:** Functional genes predicted in Longgeng 57 comparing with those from databases.

Databases	Count	Percentage (%)
SwissProt	21346	53.5
GO	21515	53.9
KO	8123	20.4
KEGG PATHWAY	5277	13.2
NR	38111	95.5
NT	39240	98.3
PFAM	21951	55.0
eggNOG	18352	46.0
Total_anno	39877	99.9
Total_unigene	39920	100.0

**Table 6:** Repeats predicted by different methods in Longgeng 57 assembly.

Type	Repeat length(bp)	% of genome
RepeatMasker	170634399	43.13%
ProteinMask	51165	0.01%
Denovo	181894436	45.97%
Trf	18598807	4.70%
Total	204035399	51.57%

**Table 7:** Non-coding RNAs annotation results in Longgeng 57 assembly.

Class	Type	Copy	Average length(bp)	Total length(bp)	% of genome
miRNA	miRNA	9684	201.5	1951247	49.3%
tRNA	tRNA	3039	75.3	228859	5.8%
	18 S	342	1739.2	594807	15.0%
rRNA	28 S	1317	144.5	190334	4.8%
	5.8 S	324	158.9	51483	1.3%
	5 S	1014	119.3	120950	3.1%
	CD-box	562	106.9	60112	1.5%
snRNA	HACA-box	64	130.2	8333	0.2%
	splicing	85	147.8	12566	0.3%

**Table 8:** Annotation results of coding region in Longgeng 57 assembly in comparing to the commonly used assemblies.

Data set	Number of proteins	Averaged gene length(bp)	Averaged cds length(bp)	Averaged exon length(bp)	Averaged intron length(bp)
MH63RS-3	60171	2610.38	1082.45	263.7	493.1
IRGSPv1.0	32441	2324.02	1047.75	264.1	431.14
ZS97RS-3	59737	2599.92	1093.13	267.86	490.07
Longgeng 57	39920	3291.76	1150.31	239.44	563.93

For the SRS data of the three parents (LN2, PN8, and LG29), we firstly aligned them against reference genome IRGSPv1.0 to gain the genome variations. Then we adopted sequences of three representative types of major genes from IRGSPv1.0 as queries and BLAST against LG57 assembly to get target sequences.

More details about data validation cases from three key genes for LG57 breeding works based on the pedigree genome data especially the assembly data of LG57 and the alignment data of its three parents were listed in Table 9. The maturing time of *Geng*/*japonica* is largely affected by *Hd1* gene^43^, which commonly harbors highly-diverse variation panels in rice genome^44^. In this region, LG57 and its three early *Geng*/*japonica* parents show extremely high consistency. The grain quality of glutinous rice is mainly controlled by *Waxy* gene^45^. LG57 possess better grain quality than other glutinous early *Geng*/*japonica* varieties, such as PN2 and LN2. There are three differences in the *Waxy* genes found between PN8 and LN2. Although a common variation in the 5^th^ exon of *Waxy* was found in PN8, LN2, and their progeny, LG57, there is a unique 23 bp deletion in the 1^st^ exon that is shared by LG57 and its non-glutinous parent, LG29. Variations in major gene *OsNramp5* affects the mineral concentrations in rice^10^. It’s notable that LG57 has variations that are different from all three parents, which is supposed to be caused by spontaneous mutations in breeding process^46,47^. Three types of variations in three representative genes validated the genome data and indicated the possible applications with this dataset. In a word, the quality of the pedigree genome data of LG57 was sufficient for public reuse in the future.

**Table 9:** Genome variations in three representative types of genes (*Hd1* for maturing time, *Waxy* for amylose content, and *OsNramp5* for mineral concentration, where 0 represents the genotype of the reference genome^36^ and 1 represents the first alternative genotype (ALT).

Target Loci	Position (bp)	Region	Ref	Alt	LG57	LN2	PN8	LG29
*Hd1*	9336605	1st exon	—	GAA insert	1/1	1/1	1/1	1/1
	9336784	1st exon	GC	AA	1/1	1/1	1/1	1/1
	9336855	1st exon	C	del	0/0	0/0	0/0	0/0
	9336944	1st exon	G	T	0/0	0/0	0/0	0/0
	9337002	1st exon	C	A	1/1	1/1	1/1	1/1
	9337005	1st exon	C	A	1/1	1/1	1/1	1/1
	9337023	1st exon	G	A	1/1	1/1	1/1	1/1
	9337038	1st exon	33 bp	de1	1/1	1/1	1/1	1/1
	9337278	1st exon	43 bp	de1	0/0	0/0	0/0	0/0
	9337404	2nd exon	TT	de1	0/0	0/0	0/0	0/0
	9337623	2nd exon	AAGA	de1	0/0	0/0	0/0	0/0
*Waxy*	1767032	1st exon	C	del	1/1	0/0	0/0	1/1
	1767036	1st exon	G	del	1/1	0/0	0/0	1/1
	1767037	1st exon	C	del	1/1	0/0	0/0	1/1
	1767039	1st exon	C	del	1/1	0/0	0/0	1/1
	1767041	1st exon	G	del	1/1	0/0	0/0	1/1
	1767044	1st exon	G	del	1/1	0/0	0/0	1/1
	1768006	5th exon	A	C, del	1/1	1/1	1/1	2/2
*OsNramp5*	8878343	2nd intron	TCTC	de1	1/1	0/0	0/0	0/0
	8872443	12th intron	A	G	1/1	0/0	0/0	0/0
	8872467	12th intron	22 bp	de1	1/1	1/1	1/1	0/0

## Data Availability

No custom code was used during this study for the curation and/or validation of the dataset.
